# Ethnic-minority health care workers discrimination: An example from Japan during COVID-19 pandemic

**DOI:** 10.7189/jogh.10.020393

**Published:** 2020-12

**Authors:** Yuki Sonoda, Yukie Matsuzaki, Masaharu Tsubokura, Yoshitake Takebayashi, Akihiko Ozaki, Hiroko Moriya, Shimmura Hiroaki

**Affiliations:** 1 Department of Nursing, Jyoban Hospital, Tokiwa Foundation, Iwaki, Fukushima, Japan; 2 Department of Public Health, Fukushima Medical University, Fukushima, Fukushima, Japan; 3 Infection Management Unit, Department of Medical Safety Management, Jyoban Hospital, Tokiwa Foundation, Iwaki, Fukushima, Japan; 4 Department of Radiation Health Management, Fukushima Medical University, Fukushima, Fukushima, Japan; 5 Department of Health Risk Communication, Fukushima Medical University, Fukushima, Fukushima, Japan; 6 Department of Breast Surgery, Jyoban Hospital, Tokiwa Foundation, Iwaki, Fukushima, Japan; 7 Department of Urology, Jyoban Hospital, Tokiwa Foundation, Iwaki, Fukushima, Japan

Infection prevention and control (IPC) in health care facilities has assumed renewed importance today as the COVID-19 pandemic continues to spread globally. While health care workers especially belong to high-risk groups of exposure and infection, the risks are not identical across all “healthcare workers”. Ethnic-minority health care workers comprise high-risk groups of exposure and infection; for example, in the UK, nine of the 10 physicians who died of COVID-19 in the early days of the pandemic were ethnic minorities. Furthermore, 63% of those who died among the main health and social care staff of the National Health Service (NHS) workforce was of BAME (Black, Asian, and minority ethnic) background [1]. Collecting sufficient information on their working styles, infection control, and countermeasures are important, not only for the safety of patients but also for their occupational health management and human rights protection.

In Japan, the number of foreign-educated nurses is increasing and they are being integrated into the medical workforce, especially by the Economic Partnership Agreements (EPA) framework, which is a regular immigration policy from Indonesia, the Philippines, and Vietnam. It is particularly necessary to identify, understand, and share minority issues that must be addressed to secure their vital position within the Japanese medical infrastructure. The authors of this research worked at Jyoban Hospital, Tokiwa Foundation in Fukushima, which is one of the Japanese medical facilities that accepts a relatively large number of Vietnamese nurses. In this article, we shared our insights on some of the challenges of the COVID-19 pandemic faced specifically by ethnic-minority medical practitioners in Japan, informed by our experiences as well as by foreign research findings. We hope that our discussion – focusing on three major topics – will help organizations to effectively utilize and manage ethnic-minority health care workers in routine clinical practice.

Our first topic is the concern that the COVID-19 pandemic is fomenting discrimination and prejudice towards ethnic-minority medical practitioners among patients and their families. Unfortunately, Vietnamese, and other foreign nurses in Japan are not unfamiliar with racial bias in in the country – a patient or family demanding a “different” primary nurse, for example – but the escalating cases of COVID-19 will be likely to encourage such racism.

Ethnic-minority health care workers have often been exposed to discrimination and prejudice, even before the current crisis: that includes verbal abuse and offensive language, not to call them by name, and to demand for the primary caregivers to switch to preferred ethnicity. According to a UK survey of general practitioners (GPs), 75% of black and ethnic-minority respondents claimed to have experienced racial discrimination from patients [2]. Even more dismaying is that these reports originated from Western nations, where demographic diversity and immigration practices have generally driven acceptance of large numbers of foreign workers in the health care workforce. To the best of our knowledge, we could locate few similar cases from Japan; however, this may simply reflect the low overall number of foreign health care workers in the country. Such unfamiliarity of many patients and families undoubtedly contributes to distrust of foreign nurses when they provide care and communicate in a clinical setting.

Stigma to certain foreigners has been observed so far during SARS and Ebola outbreak [3]. Similar to other countries heavily impacted by the pandemic, Japan is observing a rise in xenophobia and discrimination towards people from outside of Japan [4,5]. Although no foreign minorities have been reported to be infected in Japan, considering that they are routinely exposed to prejudice and discrimination, they may be pushed even further into a corner if they are reported to be infected among minorities. In fact, there have already been reports of ethnic minorities being trapped and even suicidal under COVID-19 [6].

In addition, it is worrisome that COVID-19-related fears and anxieties are serving to justify, consciously or unconsciously, discrimination towards ethnic-minority health care providers. Such prejudice – if widespread – could interfere with optimal service provision in health care settings. Hospital administrators need to recognize that this group is now at an increased risk for persecution and actions are to be taken to protect them accordingly. It is necessary to explain the infection countermeasures of medical staff to patients and their families in advance, and to protect ethnic minority health care workers by reducing patients’ anxiety.

**Figure:**
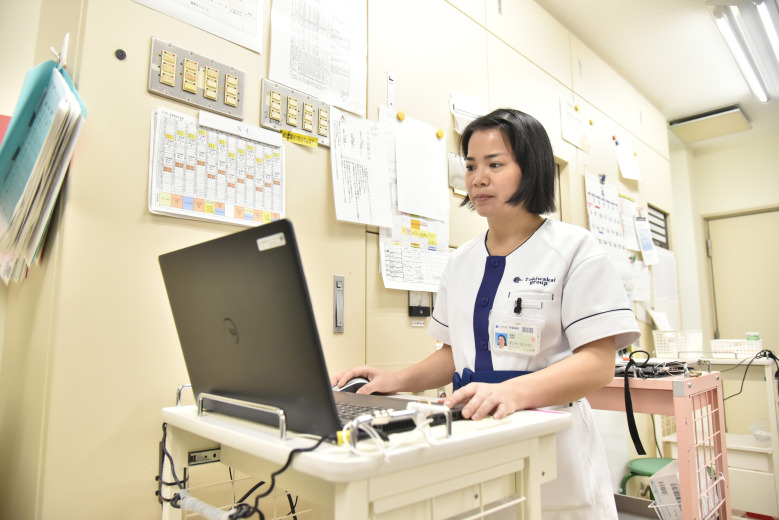
Photo: From the authors’ own collection, used with permission.

The second topic is workplace discrimination, which can leave ethnic-minority providers inadequately protected against virus transmission.

The British Medical Association (BMA) announced that ethnic-minority health care providers experience particularly high incidences of SARS-CoV-2 infection in hospitals [1]. Supply shortages of personal protective equipment (PPE eg, goggles, N95 masks, disposable gowns) – or, rather, situations where an infection occurred in the course of their duties as a result of insufficient protection – are purported to be one contributor to COVID-19 mortality among health care workers [7,8].

Low expectations from other medical staff – underestimating or even dismissing their competence – can make the workplace unreceptive to the suggestions of ethnic-minority health care providers. Even when a problem is noticed, they may shun a proactive approach to avoid discrimination and abuse [9]. In a survey conducted by the BMA, ethnic-minority GPs were almost twice as likely to claim that they lacked confidence” to raise concerns about workplace safety, fearing unfair criticism, and negative consequences if they did so.

Such malicious behavior could easily escalate into diplomatic incidents, if perceived as being contrary to official pledges to encourage the immigration of nursing candidates from other Asian countries in Japan, as stipulated in EPA. Therefore, the manager of the concerned hospital may treat foreigner nurses protectively, considering the risk of manifestation of such problems; however, we must be mindful of their risk engaging in dangerous procedures without full infection-protection measures in place, and recognize their tendency to hesitate about voicing their opinions because of workplace bullying or discrimination. Facility managers need to understand the importance of combating bias and stigma related not only to influence COVID-19 and work to eliminate discrimination and prejudice [10]. They should consciously provide accurate information not only to ethnic minority nurses who are more likely to face exclusion because of their language, but also to patients and local people. Furthermore, managers must be willing to interact with them to prevent discrimination, and it will be an effective measure against prejudice among staff.

Our third and final topic is that the language barrier may underestimate the clinical competence of ethnical minority health care workers, leading to a possible loss of appropriate vocational training opportunities.

In Japan, ethnic-minority health care workers tend not to be assigned to treating or caring for patients with COVID-19 infection or who are at high risk for it; in fact, no ethnic minorities are reported among the official death count for health care workers in Japan. This is strikingly different from the UK, where 63% of the roughly 106 health care providers who died of COVID-19 were members of an ethnic minority [9].

One possible reason for the lower number of foreign nurses on COVID-19 treatment might be the low absolute number of ethnic-minority providers working in Japanese health care, and the even smaller number working in acute care hospitals to which its patients are typically admitted. Additionally, managers tend to have less experience managing foreign-educated than native subordinates, which can make them hesitate to assign them in emergencies.

This language barrier makes many native Japanese personnel reluctant to deploy their ethnic-minority peers in urgent care situations despite their professional certification(s), knowledge, and skills. During the current pandemic, such behavior could protect them from SARS-CoV-2 infection. However, in normal practice, failing to undergo suitable workplace training for certain job aspects (eg, night shifts, measures of sudden deterioration) means a shortage of trained staff, which, especially during an emergency response, can complicate the provision of medical care.

This persistent issue has been noted long before the outbreak of COVID-19. Black and ethnic-minority workers account for 19% of the health care providers of England’s NHS and 16.4% of health care personnel in the United States of America. In Japan, on the other hand, ethnic minorities have a much smaller presence, occupying a mere 0.037% of the nursing workforce [9]. In any case, discrimination-based barriers block foreign nurses from acquiring the expert skills needed to work at the required or desired level, limiting their ability to perform their professional roles in society as well as career advancement [10].

However, with the enactment of the Japanese Language Education Promotion Act in June 2019, employers are now responsible for providing opportunities to learn Japanese for the foreign staff they employ. In anticipation of greater numbers in the future, we believe that ethnic-minority workers should be provided with ample opportunities for workplace training, including Japanese language study and case assignment, achievable by establishing or consolidating systems designed for these purposes.

The spread of COVID-19 poses a serious risk of exacerbating many long-standing issues routinely encountered by ethnic-minority health care workers in clinical practice. Racism and prejudice can be justified by fears of widespread viral transmission. These trends are undoubtedly disadvantageous to ethnic-minority practitioners in the short term, robbing them of their motivation to continue work and exacerbate high turnover rates. In the medium and long term, such disadvantages could lead to labor shortages. Thus, all necessary measures must be taken to ensure that they can provide patient care and services safely, to the same standards expected from the Japanese staff. Managers need to educate workplace colleagues, patients, and families of the unique position in which the pandemic has put ethnic-minority providers. In addition to the efforts of facility managers, the Japanese government needs to urgently enact legal measures to prevent racial discrimination so that they can safely provide patient care and services.
